# A NF-κB-Dependent Dual Promoter-Enhancer Initiates the Lipopolysaccharide-Mediated Transcriptional Activation of the Chicken Lysozyme in Macrophages

**DOI:** 10.1371/journal.pone.0059389

**Published:** 2013-03-22

**Authors:** James Witham, Lylia Ouboussad, Pascal F. Lefevre

**Affiliations:** Section of Experimental Haematology, Leeds Institute of Molecular Medicine, University of Leeds, Wellcome Trust Brenner Building, St. James’s University Hospital, Leeds, United Kingdom; Chang Gung University, Taiwan

## Abstract

The transcriptional activation of the chicken lysozyme gene (c*Lys*) by lipopolysaccharide (LPS) in macrophages is dependent on transcription of a LPS-Inducible Non-Coding RNA (LINoCR) triggering eviction of the CCCTC-binding factor (CTCF) from a negative regulatory element upstream of the lysozyme transcription start site. LINoCR is transcribed from a promoter originally characterized as a hormone response enhancer in the oviduct. Herein, we report the characterization of this cis-regulatory element (CRE). In activated macrophages, a 60 bp region bound by NF-κB, AP1 and C/EBPβ controls this CRE, which is strictly dependent on NF-κB binding for its activity in luciferase assays. Moreover, the serine/threonine kinase IKKα, known to be recruited by NF-κB to NF-κB-dependent genes is found at the CRE and within the transcribing regions of both *cLys* and LINoCR. Such repartition suggests a simultaneous promoter and enhancer activity of this CRE, initiating *cLys* transcriptional activation and driving CTCF eviction. This recruitment was transient despite persistence of both *cLys* transcription and NF-κB binding to the CRE. Finally, comparing *cLys* with other LPS-inducible genes indicates that IKKα detection within transcribing regions can be correlated with the presence of the elongating form of RNA polymerase II or concentrated in the 3′ end of the gene.

## Introduction

**Figure 1:**
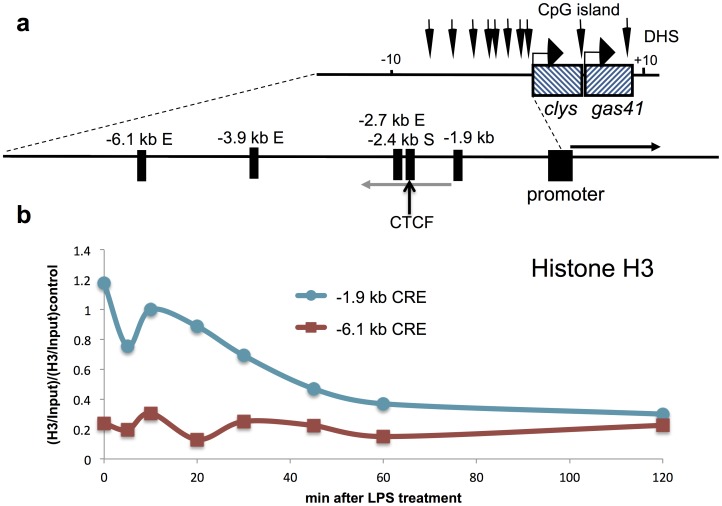
Rapid nucleosome loss at the−1.9 kb CRE in macrophages after LPS treatment. (**a**) Illustration of the chicken lysozyme locus including cis-regulatory elements and the approximate location of LINoCR transcript, grey arrow and *cLys* mRNA, black arrow [7]. (**b**) ChIP assay performed with anti-histone H3 antibody at the −1.9 kb element compared to the −6.1 kb enhancer after LPS treatment in macrophages. Horizontal axis represents time after LPS induction in minutes. Data are normalised versus input and then versus a positive control region. Data are representative of at least three independent experiments.

Genes transcription is controlled by CREs, which are, when activated, nucleosome free regions occupied by transcription factors and identified *in vivo* as DNAse I hypersensitives sites (DHS) [1]. A classical view separates these elements into different categories depending on their position from the transcription start site (TSS) of genes, their sequence and their chromatin signature. However, global analyses of the transcriptome suggest that the function of these CREs is not commonly restricted to a single category, genomic regions with dual promoter and enhancer activities appearing to be widespread within the genome. For example, a recent study looking at transcription sites located outside protein-coding regions in macrophages activated by endotoxins, found 70% of extragenic RNA polymerase II (RNAPII) peaks associated with genomic regions with a chromatin signature of enhancers [2], these elements generating very low abundance non-coding transcripts, suggested to be “junk” RNA. However, the idea that “enhancer-associated” extragenic transcription would represent only noise has already been challenged by several studies. Extragenic transcripts within locus control regions (LCR), these distal regions composed of several CREs able to enhance the expression of linked genes to physiological levels in a tissue-specific and copy number-dependent manner, have been identified some time ago and are believed to play a role in the chromatin remodelling observed over these regions [3]–[5]. More recently, the link between non-coding RNA transcription from dual promoter/enhancer elements and chromatin remodelling has been established for two chicken genes *mim-1* and *cLys* [6], [7]. However these studies did not determine if these CREs are behaving simultaneously or successively as promoter and enhancer. *CLys* is a marker of macrophage differentiation, which rapidly responds to pro-inflammatory agents like LPS and its expression is controlled by three enhancer elements situated −6.1 kb, −3.9 kb and −2.7 kb upstream of the transcription start site, a complex promoter and a silencer element at −2.4 kb (figure 1A) [8]. We have reported that *cLys* expression activation was preceded by the transient transcription of LINoCR from a promoter −1.9 kb upstream of *cLys* TSS, this transcription being necessary for nucleosome reorganisation and eviction of the enhancer blocker protein CTCF from the silencer element [7]. Interestingly, this promoter was originally identified as a hormone response enhancer element functional in the oviduct and bound by estrogen, glucocorticoid and progesterone receptors [9], [10]. However, we did not fully establish that this −1.9 kb element was driving any enhancer activity in macrophages.

In these cells, activation of the −1.9 kb element and subsequent transcription of LINoCR was correlated with accumulation of the protein kinase IKKα and histone H3 serine 10 phosphorylation (H3S10p) within LINoCR transcribed region [7]. IKKα is part of the IKK complex controlling the release of the NF-κB transcription factor into the nucleus to stimulate transcription of its target genes in response to pro-inflammatory stimuli. In the nucleus, NF-κB interacts with several chromatin modifiers including the histone H3K4 methyltransferase Set7/9 [11], CBP/p300 [12], TIP60 [13], [14] and also IKKα for which specific chromatin modifying activity has been described [15]–[17]. At the promoter of NF-κB-dependent genes, IKKα phosphorylation of H3S10 is important to initiate transcription elongation [15], [16], [18]. Furthermore, IKKα binds to the phosphorylated RNA polymerase II C-Terminal Domain (RNAPII CTD) to target HP1γserine 93 (HP1γS93) [17]. HP1γ is part of the HP1 family of proteins together with HP1α and HP1β in vertebrates. HP1 proteins are commonly associated with heterochromatin formation, as they are recruited to methylated lysine 9 of histone H3. They can in turn recruit methyltransferase to propagate silencing marks along chromatin [19], [20]. In contrast to HP1α and HP1β, HP1γ is also located in euchromatin [21] where it can be found phosphorylated at serine 93 [22], a post-translational modification abrogating the transcriptionally repressive function of HP1γ [23]. HP1γ also interacts with the phosphorylated RNAPII CTD and recruits the FACT (FAcilitates Chromatin Transcription) complex to RNAPII [24], [25]. At NF-κB-dependent genes, HP1γ controls IKKα association with chromatin and IKKα-dependent phosphorylation of the histone H3.3S31 [17].

To clarify the function of the −1.9 kb CRE, we undertook a detailed characterization of this element in macrophages. We identified a unique 60 bp region within a larger DNase I and Micrococcal Nuclease (MNase) hypersensitive domain occupied by AP1, C/EBPβ and NF-κB (p65) transcription factors after LPS treatment, the latter providing a rationale for IKKα detection within the coding region of LINoCR [7]. These transcription factors act cooperatively to fully activate this CRE. Additional luciferase reporter assays and *in vivo* chromatin immunoprecipitation analyses reveal that this 60 bp transcription factor cluster possesses concomitant promoter and enhancer activities. In addition, in contrast with what we have described for other NF-κB-dependent genes [17], IKKα is transiently recruited to both LINoCR and *cLys* transcribed regions. This loss of IKKα concomitant with LINoCR transcriptional inactivation is the only change identified at the −1.9 kb CRE, for which the chromatin structure and the transcription factors occupancy are still identical during or after LINoCR transcription. This result suggests an important role of IKKα to mediate the −1.9 kb CRE promoter activity and that the −1.9 kb CRE is important to initiate transcription of *cLys* but not to maintain this expression after CTCF eviction from the −2.4 kb silencer. This observation is linked with HP1γ poor incorporation into transcribing chromatin at *cLys* locus. These data may provide a paradigm for the modus operandi of CREs with dual promoter/enhancer activity and reinforce the idea that HP1γ controls IKKα associated transcription.

## Materials and Methods

### Cell Culture

The chicken cell lines monocytes HD11 [26] and erythroblasts HD37 [27] and the mouse cell line RAW264.7 were grown in Dulbecco’s modified Eagle’s medium as previously described [17], [28]. Mouse primary macrophages were obtained from bone marrow by culturing in Iscove's Modified Dulbecco’s Medium containing 10% Foetal Calf Serum and Penicillin-Streptomycin and 10% L cell conditioned medium containing M-CSF [29] for 7 days. Where indicated, cells were treated with 1 µg/ml LPS (Sigma).

### Chromatin Immunoprecipitation Assays and Real-Time PCR Analysis

ChIP was performed exactly as previously described [7], using dynabeads protein G (Invitrogen) with 2.4 µg per 10 µl beads of anti-p65 (Santa Cruz sc-372X), anti-Histone H3 (Abcam ab1791), anti-HP1γ (Millipore 05–690), anti-IKKα (Santa Cruz sc-7606X), anti-RNAPII S2p (Abcam ab5095) and anti-RNAPII CTD (Abcam, ab817). The primers used for the Real-Time PCR are listed in Table S1.

### Nucleosome Mapping by Indirect End Labeling

DNase I treatment of cells and naked DNA was performed as described previously [28]. MNase digestions of HD11 and indirect end labeling were performed using isolated nuclei as described previously [30]. With 10 µg of each, different DNA preparations digested with 20 U SphI (New England Biolabs) for 3 hr at 37°C and stopped with 5 µl loading dye 20% Ficoll (Sigma), 1% SDS (Sigma), and 0.05% bromophenol blue (Sigma). The probe abutting the SphI site (−3165 to −2865 bp) was prepared by PCR using a plasmid containing the full sequence of the lysozyme locus as a template with the following primers: fwd, TACTTAGGAGGGTGTGTGTG and rev, GCACCTTGAAGATTTGTT. The probe was gel purified using a QIAquick Gel Extraction Kit (QIAGEN). Bands were quantified from the images generated on the pharosFX molecular imager using Quantity One software (BioRad).

### DMS In vivo Footprinting Analysis

7.5×10^6^ HD11 cells were seeded in a 10 cm dish and incubated overnight before they were treated with LPS. *In vivo* footprinting has been performed as previously described [31] with the following primers: non-coding strand (A biotin-GGGTTAGTAATGTTAATCTCA, B AGAAGCCAACCCTGACAGACATC, C ACAGACATCCCAGCTCAGGTGGAAATC and the respective annealing temperatures 53°C, 60°C, 66°C), coding strand (A biotin-CACTCCCTGACCATAGCT, B GGAACTTCTGCTCCTTGGATCAC, C TTGGATCACGGCCTGACCCAAAAAGT and the respective annealing temperatures 53°C, 60°C, 66°C), figure S1A (A biotin-CCTCAAGGTAACTGATGTT, B CAGAGGCAATCCTGGAATTTTCTC, C CCTGGAATTTTCTCTCCGCTGCACAGTT and the respective annealing temperatures 51°C, 54°C, 67°C), figure S1B (A biotin-ACTTGCTGAGGATTAATGT, B TGCAATTTCAACAAAAGCCACTCT, C CAAAGGCGAAACCACAAGAGTGGCTTTT and the respective annealing temperatures 51°C, 62°C, 67°C).

### Cloning, Mutagenesis and Transient Transfection

DNA fragments carrying the lysozyme promoter (−376 to +17 bp) and the 1.9 kb element (−2132 to −1877 bp) were cloned into the luciferase vector pXPG [32]. Mutants were generated by PCR amplification in the following 50 µl reaction mixtures: 1X Pfu Turbo buffer (Stratagene), pXPG-1.9AS [7] as a template, 125 ng of both forward and reverse primers, 0.25 mM dNTPs and 2.5 U Pfu Turbo (Stratagene). PCR amplification conditions were as follows: (1) denaturation at 95°C for 30 sec, (2) 16 cycles of denaturation at 95°C for 50 sec, annealing at 55°C for 50 sec and extension at 72°C for 7 min and (3) a final extension at 72°C for 7 min. Next 1 µl of 20 U/µl DpnI (NEB) was added directly to the PCR mix, incubated for 80 min at 37°C and then heat inactivated by incubation at 80°C for 20 min. Then 50 µl of stable 3 electro-competent bacteria (Invitrogen) were transformed, incubated and plated according to manufacturer recommendations. Transfection and luciferase assays were performed as previously described [7].

### Preparation of Nuclear Extracts and Electrophoretic Mobility Shift Assay

2×10^7^ HD11 cells, unstimulated or stimulated with 1 µg/ml of LPS for 1 hr and nuclear extracts prepared as previously described [33]. EMSAs were performed using end-labelled, double-stranded synthetic oligonucleotides. 2 µg of nuclear extracts was diluted in EMSA buffer [33]. The buffer-diluted samples then formed complexes with either 50 ng of unlabelled competitors or 1 µg specific antibodies, anti-c-FOS (Sc-253, Santa Cruz) and anti-C/EBPα (Sc-61, Santa Cruz) during an incubation for 15 min at 25°C before the addition of 0.5 ng ^32^P γ labelled probe. After incubation with the probe for 15 min at 25°C, the samples were separated on a 5% acrylamide gel (37.5∶1), 0.5 X TBE, 1/1000 TEMED and 0.1% APS. The gel was fixed, dried, exposed with a K-Screens (KODAK) for 16 hrs and analysed on pharosFX molecular imager (Biorad).

## Results

### Identification of a 60 bp Region within the **−**1.9 kb CRE Occupied by Transcription Factors after LPS Treatment in Macrophages

**Figure 2:**
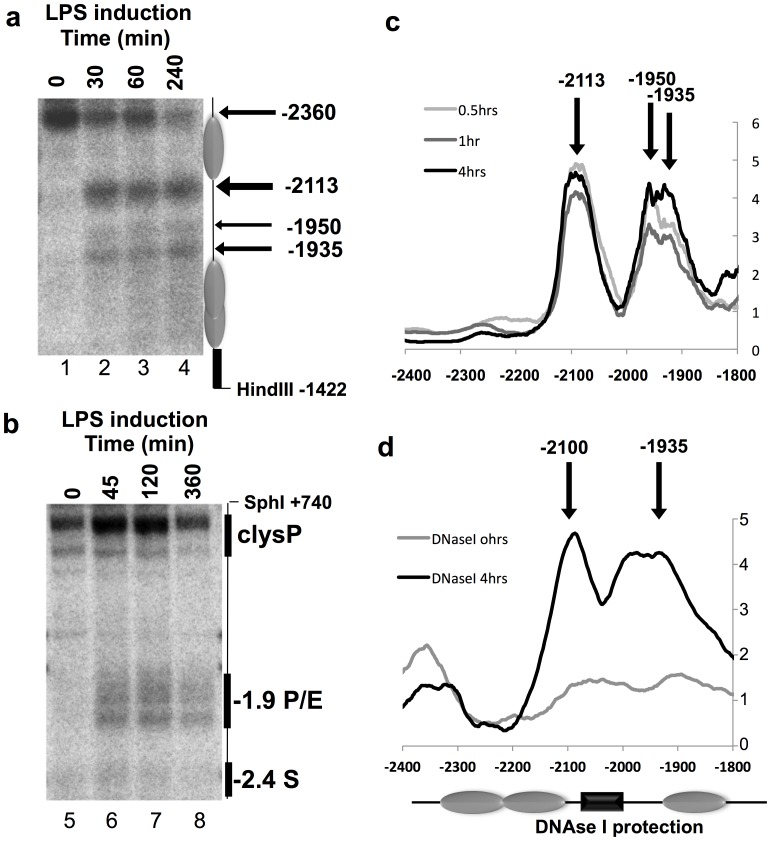
*In vivo* MNase and DNase I mapping of the −1.9 kb element reveal chromatin remodelling in response to LPS. Southern blot of (**a**) MNase or (**b**) DNAse I digested genomic DNA from permeabilised nuclei of unstimulated HD11 (lane 1 and 5) or HD11 LPS (5 µg/ml) stimulated (**a**) for 30 min, 60 min and 240 min (lanes 2–4) or (**b**) for 45 min, 120 min and 360 min (lanes 6–8). Inferred nucleosome positions are illustrated in grey. Width of arrows indicates the degree of cleavage and the approximate position relative to the *clys* transcription start site. These experiments are representative of two independent experiments. Quantification of (**c**) the MNase, LPS stimulated samples are normalised to unstimulated HD11 and (**d**) the DNAse I southern blots. Arrows are indicating approximate position of cleavages. DNAse I protection domain within the −1.9 Kb element is illustrated by a black box.

We have previously established that the eviction of the insulator-associated protein CTCF from its binding site and subsequent nucleosome movement over this site was dependent on transcription of LINoCR at the *cLys* locus in activated macrophages [7]. LINoCR is firing from a CRE located −1.9 kb upstream of *cLys* TSS, this element being previously described as a hormone response enhancer in the oviduct [9], [10] (figure 1a). These observations were making this CRE, one of the first characterized dual promoter/enhancer for which the associated transcription of the non-coding RNA was known to be functional. However, our previous work did not clearly establish that this element was also acting as an enhancer in macrophages. To determine, if this CRE could represent a paradigm of dual promoter/enhancer elements or if, in macrophages, it was only a promoter, we decided to undertake a more detailed characterization of this element. First, we looked at the nucleosome content in this region before and after LPS treatment in the chicken macrophage cell line HD11 compared to the −6.1 kb enhancer by chromatin immunoprecipitation (ChIP) using an antibody against total histone H3. We detected a progressive reduction in nucleosome content starting 20 min after LPS treatment and reaching a plateau after 45 min (figure 1b). Then, we performed low resolution DNase I hypersensitive site (DHS) and nucleosome (MNase) mapping analyses in response to LPS in the HD11 cell line (figure 2). Both MNase and DNase I mapping revealed a large region between −1.9 kb and −2.1 kb, becoming nucleosome free and DNase I accessible as early as 30 min post LPS stimulation (figures 2a and 2b). Additional quantification of these two southern blots indicated that within the 200 bp delimiting the −1.9 kb element a region between 60 to 100 bp was more protected against DNase I and MNase (figures 2c and 2d). Taken together, these results confirm that the −1.9 kb element is inactivated in absence of pro-inflammatory stimuli and highlight a small region within this element protected from MNase and DNase I digestions.

**Figure 3:**
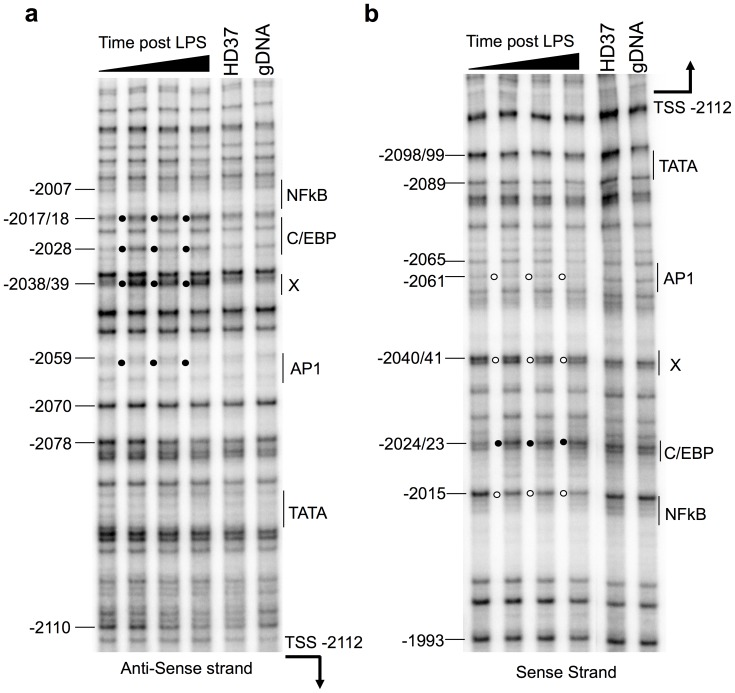
*In vivo* DMS footprinting of the −2000 to −2150 bp region, part of the −1.9 kb CRE, reveals transcription factor occupancy in response to LPS stimulation. HD11 cells were, in order from left to right, either unstimulated or LPS (1 µg/ml) stimulated for 30 min, 60 min or 240 min. Cells were then treated with DMS before the isolation of genomic DNA for hot piperidine cleavage and LM-PCR analysis. The HD37 erythroid cell line which do not express *clys* and the naked HD11 genomic DNA, G reaction, reference sequence are also shown. (a) non-coding strand and (b) coding strand. The filled circles represent DMS hyper-methylation and the open circles base protection from DMS. The positions of the selected G bases are indicated relative to the *cLys* transcription start site. The potential transcription factors are indicated adjacent to a single line encompassing their proposed binding site. LINoCR transcription start site is indicated with an arrow. These images are representative of the result of two independent experiments.

To map precisely where transcription factors bind in this element, we undertook some Dimethyl Sulphate (DMS) *in vivo* footprinting analysis. A first set of primers provided information on transcription factors occupancy in the −2150 bp to −2000 bp region (figure 3). In untreated HD11 cells, the DMS modification pattern on both strands was indistinguishable from the non-expressing HD37 and the G-reaction performed on naked HD11 genomic DNA. This confirms that myeloid specific transcription factors did not occupy the upstream region of the −1.9 kb element prior to LPS activation. Immediately post LPS stimulation, there was hyper-reactivity of Guanine bases at three positions, −2017/18, −2028 and −2038/39 bp on the anti-sense strand (figure 3a). All three footprints were present within 30 min and remained throughout the 240 min time course. Analysis of the sense strand confirmed these observations. LPS activation produced differential hypersensitivity at three sites −2040/41, −2024/23 and −2015 bp (figure 3b). However, on the sense strand, two Guanine bases, at −2040/41 and −2015 bp, were protected and one, at −2024/23 bp, was hyper-reactive. Sequence analysis associated a consensus-binding site for NF-κB between −2015 and −2017/18 bp and for C/EBP between −2017/18 and −2028 bp. Further upstream, although the footprint was weak, there were indications of transcription factor binding on the sense strand at −2061 and −2065 bp. The sequence surrounding this footprint revealed a potential AP1 binding site. This is consistent with the characteristic weak footprints of AP1 factors and with previous ChIP experiments detecting Fos binding to the −1.9 kb region immediately post LPS stimulation [7]. A second set of primers allowed to analyse the transcription factor occupancy post LPS stimulation of the −2000 to −1850 bp region. However, apart from the previously identified potential C/EBP and NF-κB binding sites we did not detect any reproducible DMS footprints in this region (figure S1). Having analysed the entire nucleosome free region of the −1.9 kb element it was clear that the key transcription factors were clustered within 60 bp in the upstream section of the DHS. In addition, the low-resolution analyses confirmed that the DHS and MNase sensitive domain represent the region immediately upstream of LINoCR and therefore the promoter of this ncRNA, our previous experiments having identified LINoCR TSS −2.12 kb upstream of *cLys* TSS [7]. Further inspection of the upstream sequence of the proposed transcription factor binding site cluster revealed a non-classical TATA box, TACATAAA, located 21 bp from the proposed AP1 binding site [34]. In summary, the DMS footprints implicate AP1, C/EBP and NF-κB binding to sites in the upstream region of the −1.9 kb dual promoter enhancer element. The positioning of the transcription factor binding sites relative to the ncRNA transcription start site and the proposed TATA box were consistent with the structure of a promoter.

### NF-κB Occupies *in vivo* the **−**1.9 kb CRE and Recruits IKKα to both LINoCR and *cLys* Transcribing Regions Simultaneously

The *in vivo* DMS footprinting revealed the specific sites within the −1.9 kb element at which transcription factors were binding in response to LPS. The subsequent analysis of the sequence encompassing the footprints implied that NF-κB, C/EBP and AP1 were binding. This was confirmed by electrophoretic mobility shift assays (EMSA) (figures S2 and S3a–d). In these experiments, NF-κB appeared to bind only weakly to the −1.9 kb element. However, additional EMSA experiments with a 40 bp oligonucleotides encompassing both the proposed C/EBP and NF-κB sites showed a cooperative binding of both proteins to this CRE (figure S3e).

**Figure 4:**
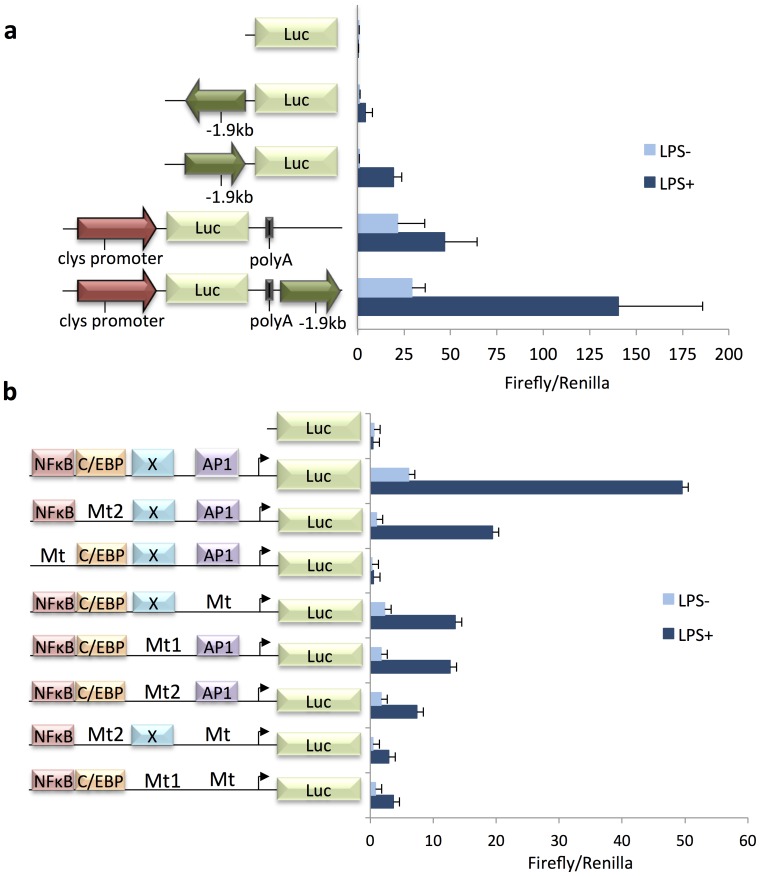
The −1.9 kb element is a NF-κB-dependent LPS inducible promoter and enhancer in transient transfection. HD11 macrophages were transfected for 18 hrs with Jetpei 2 µl and 1 µg DNA prior to 7 hrs 1 µg/ml LPS stimulation, black bars, or remained untreated for 7 hrs, grey bars. The constructs are illustrated adjacent to the y-axis. (**a**) *Clys* promoter is a black arrow, the −1.9 kb CRE is a dark grey arrow, the firefly luciferase coding region is grey. (**b**) The −1.9 kb element is cloned in antisense orientation, Position of the NF-κB, C/EBP, X unknown protein and AP1 are indicated next to the Y axis. (**a** and **b**) The data are plotted as the mean value of two independent experiments, individual experiments had triplicate samples for each condition. Inter sample variation has been corrected by Renilla normalisation. Positive error bars indicate standard deviations.

We have previously shown that the −1.9 kb CRE cloned immediately upstream of *cLys* promoter in sense orientation was increasing this promoter LPS-dependent inducibility suggesting that this CRE was an enhancer in macrophages [7]. To complete this observation, we cloned the −1.9 kb CRE downstream of the luciferase polyA signal in a *cLys* promoter driven pXPG reporter vector, to rule out the possibility that the −1.9 Kb element cloned immediately upstream of *cLys* promoter generated an extended promoter. In agreement with previous observations, LPS stimulated weakly *cLys* promoter activity (figure 4). Furthermore, in the resting HD11 cells, *cLys* promoter activity with the −1.9 kb element cloned in 3′ was equivalent to *cLys* promoter alone. However, upon LPS incubation, *cLys* promoter activity was increased 4.8 fold when the −1.9 kb element was present as opposed to 2 fold LPS induction with just *cLys* promoter (figure 4a). Thus, the −1.9 kb element enhanced the *cLys* promoter’s LPS inducibility by approximately 2.5 fold. These experiments established that the −1.9 kb element was a LPS-inducible enhancer in macrophages. The strict LPS dependence of both the −1.9 kb promoter and enhancer capabilities implies that the inflammatory response regulates the transcription factor(s) required for its activity.

Having determined the location, identity and binding ability of transcription factors present at the −1.9 kb element in the activated HD11 cells, their individual contribution to promoter activity was assessed in transient transfection. An extensive set of constructs containing the individual or combination of binding inactivation mutations, revealed by EMSAs, were cloned into the pXPG luciferase reporter plasmid and transfected into HD11 cells (figure 4b). The mutation preventing NF-κB binding to the −1.9 kb element completely abolishes both basal and LPS inducible activity of this promoter. In addition, mutations of C/EBP, X or AP1 show similar impact on this promoter’s basal and LPS-inducible expression, expression being further reduced by double mutants C/EBP and AP1 or X and AP1. Taken together, these results show that NF-κB is essential for the promoter activity of the −1.9 kb element but does not act alone as each individual mutation has a significant impact on the promoter activity in the transient transfection assays.

**Figure 5:**
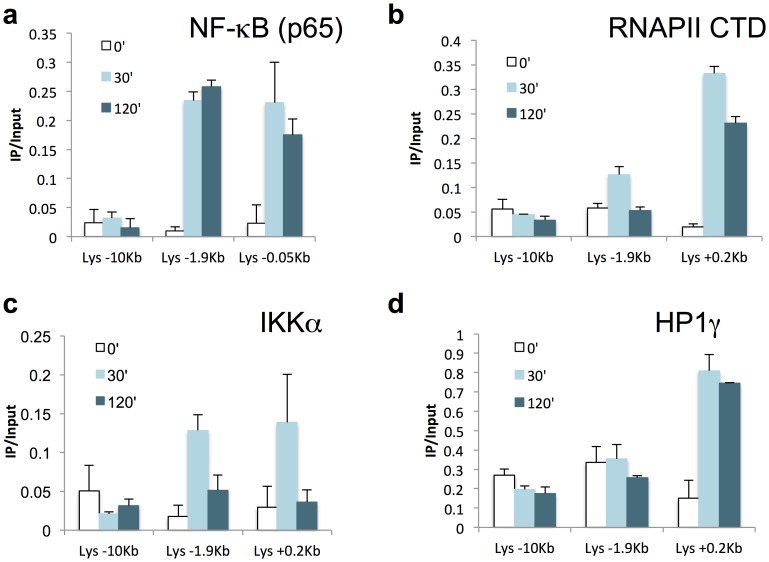
LPS induces recruitment of NF-κB, IKKα, RNAPII and HP1γ with different kinetics. (**a–d**) ChIP performed with primary macrophages treated with LPS for the indicated time points in minutes and the following antibodies (**a**) anti-p65 (NF-κB), (**b**) anti-RNAPII CTD, (**c**) anti-IKKα and (**d**) anti-HP1γ. Horizontal axis indicates primers used for the Real time PCR (distance in kb from the transcription start site of *cLys*). Data are normalized versus input. Error bars represent SD from three independent qPCR replicates. These data are representative of at least three independent experiments.

**Figure 6:**
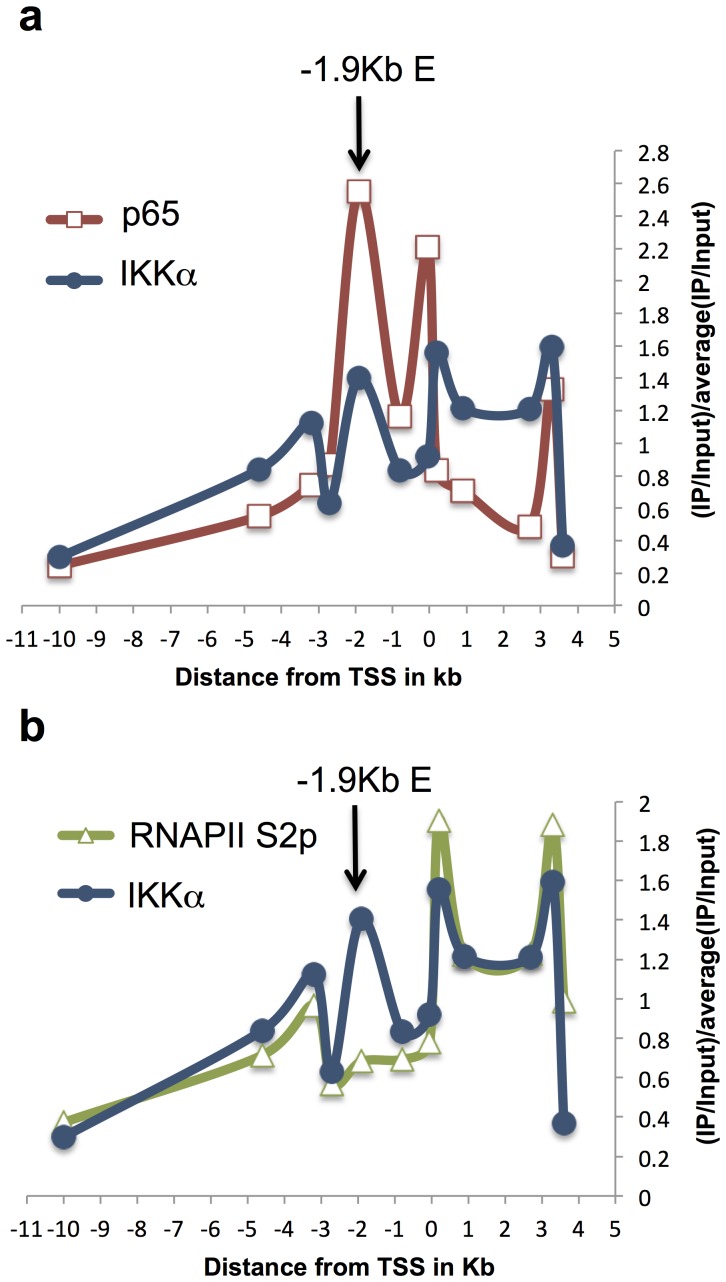
RNAPII S2p and IKKα occupancies at *cLys* locus overlap. (**a–c**) ChIP performed with primary macrophages treated with LPS for 30 min and the following antibodies (**a**) anti-p65 (NF-κB) and anti-IKKα, (**b**) anti-RNAPII S2p and anti-IKKα (**a–b**) Position of the −1.9 kb element is indicated by an arrow. Horizontal axis indicates distance from *cLys* transcription start site. Data are normalized versus input and then versus the average of all IP/Input values. These data are representative of at least three independent experiments.

We have shown previously that C/EBPβ and Fos were binding to the −1.9 kb element after LPS treatment in HD11 [7]. In this work, C/EBPβ and Fos were found enriched at the −1.9 kb element from 20 min post LPS treatment in agreement with experiments above describing a quick activation of this element in response to LPS. If this element is activated early after stimulation, we did not detect any change in *cLys* basal mRNA level before 45 min post LPS. Because detectable changes in total mRNA level are delayed compared to transcriptional activation, we could not determine if the −1.9 kb element was first acting as a promoter and then as an enhancer after LINoCR expression was stopped or if this CRE could act simultaneously as promoter and enhancer. Using a transgenic mouse line harbouring the 21 kb *cLys* domain inserted into the HPRT locus [35], we first performed additional chromatin immunoprecipitation experiments looking at the NF-κB protein family member p65 in primary macrophages. As expected we detected enrichment for p65 in both *cLys* promoter and the −1.9 kb element after 30 min and 120 min of LPS treatment (figures 5a and S4c). Interestingly if p65 binding was stable after short-term and long-term LPS treatment, total RNAPII or elongating RNAPII (RNAPII S2p) occupancies were higher in *cLys* coding region (0.2 kb to 3.6 kb) after 30 min than after 120 min of LPS treatment (figures 5b, S4a and S4b). As expected, RNAPII was only detectable within LINoCR transcribing region (−1.9 kb to −3.2 kb) after short-term LPS treatment (figures 5b, S4a and S4b). In addition, this RNAPII enrichment was correlated with IKKα recruitment to the transcribed regions of both *cLys* and LINoCR (figures 5c and S4d). More detailed comparisons between p65, IKKα and RNAPII recruitment after 30 min of LPS treatment highlight that IKKα correlates with RNAPII but not with NF-κB recruitment except at the −1.9 kb element where both IKKα and p65 but not RNAPII S2p are detected (figures 6a and 6b). This observation suggests that IKKα is recruited to *cLys* locus by NF-κB bound to the −1.9 kb element and not to *cLys* promoter. In addition, this result indicates that LINoCR and *cLys* are transcribed simultaneously and that IKKα-dependent transcription is restricted to early time points.

### IKKα Recruitment to NF-κB-dependent Genes can Follow Different Kinetics

**Figure 7:**
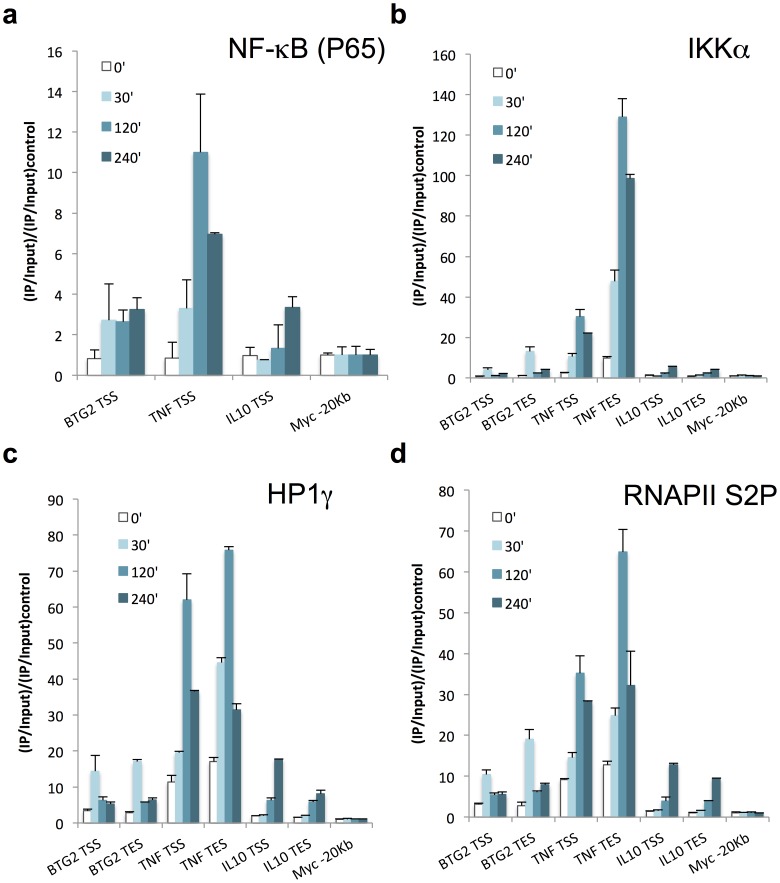
NF-κB-dependent genes show specific temporal patterns of IKKα recruitment (**a**–**d**) ChIP performed with RAW264.7 cell lines treated with LPS for the indicated time points in minutes and the following antibodies (**a**) anti-p65 (NF-κB), (**b**) anti-IKKα, (**c**) anti-HP1γ and (**d**) anti-RNAPII S2p. Horizontal axis indicates primers used for the Real time PCR. Data are normalized versus input and then versus the Myc −20 Kb control region. Error bars represent SD from three independent qPCR replicates. These data are representative of at least three independent experiments.

IKKα enrichment profile was not temporally and spatially comparable with our previous observations made for *TNF*, *Ccl3* or *Il1β* other NF-κB-dependent genes [17]. At these genes, IKKα accumulates in the 3′ end, with 10 times more IKKα detected in 3′ compared to the promoter region, binds to chromatin in an HP1γ-dependent manner and is still detectable after 2 h of LPS stimulation. We hypothesised that IKKα was not interacting with chromatin because of the poor incorporation of HP1γ to transcribing chromatin within *cLys* locus. Indeed, we detected only 3 to 4 times HP1γ enrichment in the coding region of *cLys* and no enrichment at all in LINoCR-transcribing region compared to a CTCF-binding site located upstream of the murine IL6 TSS where HP1γ was undetectable in macrophages (figures 5d and S4e). *TNF*, *Ccl3* or *Il1β* respond to LPS with similar kinetics. To complete our analysis, we chose two additional NF-κB-dependent genes with different temporal patterns of expression, *BTG2* and *IL10*, for which expression peaks before or after 2 h of LPS treatment respectively (figure S5). ChIP experiments performed 30 min, 2 h and 4 h post LPS stimulation showed p65 binding to the promoter of both genes (figure 7a). Moreover, enrichment for HP1γ correlated with RNAPII S2p at these genes in agreement with what we observed for *TNF* and *cLys* (figures 7c, 7d and S6a), indicating that the amount of HP1γ detected within the coding regions of these genes shadows the rate of transcription. The analysis of IKKα recruitment to these loci unveiled a more heterogeneous association of this kinase with the different NF-κB-dependent genes studied. At the *BTG2* promoter, the dynamics of p65 and IKKα occupancy mimicked our observation for the *cLys* −1.9 Kb CRE. In parallel with *BTG2* expression, IKKα, HP1γ and RNAPII S2p were only detected within the gene 30 min after LPS stimulation (figures 7b-c). At the 3′ end of the gene, IKKα enrichment was 4 fold higher than at the promoter whereas HP1γ was also 4 fold more associated with *BTG2* than with *cLys* but 4 fold less than with *TNF* (figures 7b and 7c). In contrast, the promoter of *IL10*, for which LPS-mediated transcription is delayed compared to *BTG2*, was not bound by p65 before 2 h post LPS stimulation (figure 7a). In addition, if p65, HP1γ and RNAPII S2p enrichments within this locus were comparable at 4 h with the one observed at *BTG2* locus after 30 min of LPS treatment, IKKα was 2.5 to 3 fold less recruited to *IL10* locus compared to the latter (figures 7a–d). These data would suggest that IKKα is mainly playing a role during the earliest stage of LPS-mediated transcription. However, we have shown previously that the presence of IKKα at transcribing NF-κB-dependent genes could be maintained after 2 h of transcription [17]. At these genes, the accumulation of IKKα downstream of the transcription end site (TES) is independent of RNAPII S2P (figure S6b). Taken together, these data highlight the fact that IKKα recruitment dynamics can obey different rules during transcription and suggest that HP1γ controls this dynamics.

## Discussion

Enhancers with a “cryptic” promoter activity are widespread along the genome, but the functions of the produced ncRNAs, if functions at all, are still unknown [2]. Concomitantly, two dual promoter/enhancer CREs have been described, for which remodelling of the surrounding chromatin domains depends on their promoter activity and associated transcription of ncRNAs [6], [7]. However in these examples, the exact dynamic of promoter and/or enhancer activity during transcription of the associated protein-coding gene is still unclear. For example, *cLys* −1.9 kb CRE promoter and enhancer activities have been documented but in different cell types, enhancer in the oviduct and promoter in macrophages [7], [9], [10]. In this study, we determined that the −1.9 kb CRE is a LPS-responsive element in macrophages controlled by a unique 60 bp transcription factors cluster occupied by AP1, C/EBP, NF-κB and a still unidentified factor, additional ChIP experiments confirming that Fos, C/EBPβ [7] and NF-κB (p65) were binding *in vivo* to this CRE. The key inflammatory factor NF-κB is the main regulator of this CRE, which does not show any activity in absence of NF-κB binding in luciferase assays. If NF-κB is necessary, AP1, C/EBP and an undetermined factor act synergistically with NF-κB to provide full activity of this element. Such cooperative function between AP1, C/EBPβ and NF-κB has been described for multiple LPS-inducible genes including IL-6, CXCL8 or IL-8 [36]–[38]. *In vitro*, NF-κB binding is stronger in presence of C/EBP bound next to its binding site, like observed for IL-6 and IL-8 promoters, where C/EBPβ and NF-κB directly associate with each other [39]. In transient transfection, C/EBP mutant alters only slightly the −1.9 kb promoter activity suggesting that C/EBP-independent binding of NF-κB is stronger *in vivo* compared to EMSA or that the alteration of the −1.9 kb promoter activity in C/EBP mutant is mainly caused by the reduction of NF-κB binding. The fact that *cLys* contains three functional C/EBP binding sites bound by C/EBPβ [7], [40] and shows only a slight increase in activity after LPS treatment reinforce the second hypothesis.

This cluster of LPS-inducible transcription factors is distinct from the steroid receptor binding sites involved in regulating this CRE in the oviduct [9] and from the mapped progesterone transcription factor binding sites, which are at a greater distance from LINoCR TSS [41], but still within the 200 bp nucleosome free region. These differences would argue that this CRE accomplishes different functions in the oviduct and in macrophages. However, additional transient transfections with the −1.9 kb CRE cloned at the 3′ end of a luciferase gene driven by *cLys* promoter confirms that the same 60 bp transcription factor cluster also drives a LPS-inducible enhancer activity in macrophages. Because our previous data suggest that *cLys* transcriptional activation was a strict consequence of CTCF eviction induced by LINoCR, the −1.9 kb CRE was expected to act successively as a promoter and then as an enhancer consequently to LINoCR expression being turned off. Surprisingly, this is not the model this study is revealing. ChIP experiments show p65 binding to *cLys* promoter and the −1.9 kb CRE after both 30 min and 120 min post LPS treatments. In contrast, IKKα is only recruited to the −1.9 kb CRE and not to *cLys* promoter and this recruitment is seen only after the shortest period of LPS treatment. The absence of IKKα at *cLys* promoter could be explained by the fact that the NF-κB heterodimer p65:c-Rel and not p65:p50 occupies *cLys* promoter [42]. If p65:p50 dimer recruits IKKα to the promoter of NF-κB-dependent genes [15], [16], p65:c-Rel has been shown to recruit IκBβ to a selected group of genes in response to LPS including *TNF* and *IL1β* [43], [44]. Furthermore, IKKα is detected in both transcribing regions probably bound to the phosphorylated RNAPII CTD as reported previously [17]. This observation suggests a direct connection between *cLys* promoter and the −1.9 kb CRE and simultaneous promoter and enhancer activities of this element. This contact could be mediated by C/EBPβ, which has been shown to form long rang interaction and DNA looping [45]. If the short distance between these two elements does not allow chromosome conformation capture (3C) analysis, the hypothesis of a direct interaction is reinforced by recent views regarding the chromatin organisation in euchromatin. These two nucleosome free regions should be indeed physically in close proximity within the 30 nm chromatin fiber structure, which has been shown to be conserved at transcribing regions [46].

C*Lys* expression is maintained several hours after that *LINoCR* has been shut down [7], but this expression is IKKα independent. The reasons, for IKKα disappearance from *cLys* locus, are unclear since the transcription factors and especially NF-κB (p65) still occupy their binding sites at the −1.9 kb CRE several hours post LPS treatment. It could be explained by the inactivation of the transcription factors, bound to this regulatory element, by post-translational modifications. For example, p65 can be activated by Msk1-dependent phosphorylation of its serine 276 and deactivated by PP2A phosphatase without affecting p65 DNA binding [47], [48]. Post-translational modifications also regulate C/EBPβ activity [49]–[51], C/EBPβ preventing NF-κB phosphorylation and thus its activation in TNF tolerant cells [52]. Finally, the composition of the AP1 dimer alters the transcriptional activation capability of this transcription factor [53]. Together, these results suggest that the transcription factors bound cluster within the −1.9 kb CRE can be inactivated without observable changes in DNA binding. In this model, the −1.9 kb CRE would initiate transcription of *cLys* and would concomitantly abrogate CTCF enhancer blocker activity. The maintained expression of *cLys* would thereafter be controlled by the three-enhancer elements upstream of CTCF, the −1.9 kb element playing a minor role or no role at all after CTCF eviction. Such a transient role of IKKα is observed for *BTG2*, for which p65 binding to the promoter is maintained after loss of IKKα and in absence of transcription. However *IL10* expression, which is delayed compared to the other analysed genes, is concomitant with p65 binding to its promoter arguing for a role of this protein in transcription of late LPS responsive genes. NF-κB and IKKα binding to the IL10 promoter is induced by the HIV-1 TAT protein but observed 30 min after stimulation [54]. In contrast, when compared to *BTG2*, IKKα recruitment to IL10 locus after LPS stimulation is poor suggesting that NF-κB (p65) can activate genes independently of IKKα or that p65 is playing a minor role in IL10 expression, as suggested by other studies [55], [56]. Nevertheless, the presence of IKKα can be measurable after 15 min of LPS stimulation and maintained beyond 2 h within transcribing regions in correlation with NF-κB (p65) promoter occupancy as observed for *TNF*, *Ccl3* or *IL1β* [17]. This “extended” detection of IKKα correlates with a dense distribution of HP1γ throughout the transcribing regions of these genes [17]. As described for IKKα, HP1γ directly interacts with the elongating polymerase [24], [25] and closely mimics the distribution of the latter during transcription. In addition, chromatin accumulation of IKKα downstream of the *TNF* transcription end site in activated macrophages is HP1γ-dependent [17]. This chromatin association of IKKα is not observed for *cLys* and *BTG2* for which the distribution of this kinase with both HP1γ and RNAPII S2p are correlated.

In macrophages, the *cLys* −1.9 kb CRE is driven by a 60 bp transcription factors cluster and is a NF-κB/IKKα-bound dual promoter and enhancer with both activities being simultaneous. Its promoter activity is associated with chromatin remodelling of the transcribed region and CTCF eviction and its enhancer activity is characterised by initiation of *cLys* transcription. In addition, we have determined that IKKα association with coding regions can be restricted to the earliest stage of LPS-mediated macrophages activation, in contrast with genes, like *TNF*, which display chromatin associated IKKα in the 3′ end of the gene [17]. These two scenarios suggest a NF-κB-dependent recruitment of IKKα during the initiation step of transcription, followed for some genes by a conservation of the kinase within the transcribing locus, this conservation being possibly NF-κB independent.

## Supporting Information

Figure S1 ***In vivo*** **DMS footprinting of the distal part of the** −**1.9 kb promoter**. HD11 cells were, in order from left to right, either unstimulated or LPS (1 µg/ml) stimulated for 30 min, 60 min or 240 min. Cells were then treated with DMS before the isolation of genomic DNA for hot piperidne cleavage and LM-PCR analysis. The HD37 erythroid cell line which do not express *clys* and the naked HD11 genomic DNA, G reaction, reference sequence are also shown **(a)** non-coding strand and **(b)** coding strand. The filled circles represent DMS hyper-methylation and the open circles base protection from DMS. The positions of the selected G bases are indicated relative to the *clys* transcription start site. The potential transcription factors are indicated adjacent to a single line encompassing their proposed binding site. These images are representative of two independent experiments.
(TIFF)

Figure S2 **Sequence of the −1.9 Kb promoter/enhancer element and detailed EMSA’s probes.** (**a**) The proposed transcription factor binding sites are double lined and colour coded; the AP1 site is gold, C/EBP site is green, NF-κB site is blue and DMS footprints for X (unidentified) are purple. LINoCR transcription start site (TSS) and proposed TATA box are shaded grey. Numbers located above the sequence are base pair positions relative to the *clys* transcription start site. (**b**) Probes designed for EMSA experiments. Base pair exchanges in designed mutants (Mt) are indicated at the top of the specific sequence.
(TIFF)

Figure S3 **AP1, C/EBP and NF-κB transcription factors bind to the proposed site in the −1.9 kb CRE** ***in vitro*** **.** Electromobility Shift Assay demonstrating specific binding of (**a**) AP1, (**b**) X (unidentified), (**c**) C/EBP, (**d**) NF-κB and (**e**) C/EBP and NF-κB to the −1.9 Kb element. ^32^P labelled oligonucleotide probes were incubated with crude nuclear extract from HD11 stimulated 1 hr with LPS (1 µg/ml) or with buffer alone (lanes 1). Specific DNA:Protein complexes are indicated with arrows. The supershift, when the reaction mixture was incubated with 1 µg of anti-cFos antibody (a, Lane 5), is indicated with an asterisk (*). Probes and cold competitors (100x) or antibodies (anti-) are indicated at the top of each lane. Sequences of the probes are detailed in figure S2. These figures are representative of two independent experiments.
(TIFF)

Figure S4 **IKKα recruitment to the** ***cLys*** **locus is transient.** (**a–e**) ChIP performed with primary macrophages treated with LPS for the indicated time points in minutes and the following antibodies (**a**) anti-RNAPII CTD, (**b**) anti-RNAPII S2p, (**c**) anti-p65 (NF-κB), (**d**) anti-IKKα and (**e**) anti-HP1γ. Horizontal axis indicates primers used for the Real time PCR (distance in Kb from the transcription start site of *cLys*). Data are normalized versus input and then versus a background control region designed within a CTCF binding site at the IL6 locus. Error bars represent SD from three independent qPCR replicates. These data are representative of at least three independent experiments.
(TIFF)

Figure S5 **Changes in** ***BTG2*** **and** ***IL10*** **expression in response to LPS treatment.** Time course of *BTG2* (blue squares) and *IL10* (red squares) mRNA levels in RAW264.7 cells in response to LPS treatment. Results are expressed relative to GAPDH expression. Error bars represent SD from three independent experiments.
(TIFF)

Figure S6 **IKKα accumulates downstream of TNF TES independently of the elongating polymerase.** (**a** and **b**) scatter plots showing the degree of correlation between the elongating polymerase (Y axis) and (**a**) HP1γ (x axis) or (**b**) IKKα (x axis). Blue rectangles display values from data presented in figure 6. Red lozenges are the same values without TNF TES 30′, 120′ and 240′. Trend lines and R-squared values are display on the figures.
(TIFF)

Table S1 List of primers used in this study.
(TIFF)
