# Supplementary figures and images for: A NF-κB-Dependent Dual Promoter-Enhancer Initiates the Lipopolysaccharide-Mediated Transcriptional Activation of the Chicken Lysozyme in Macrophages
Source: PLoS One. 2013 Mar 22;8(3):e59389. doi: 10.1371/journal.pone.0059389 (PMC3606415; doi:10.1371/journal.pone.0059389)

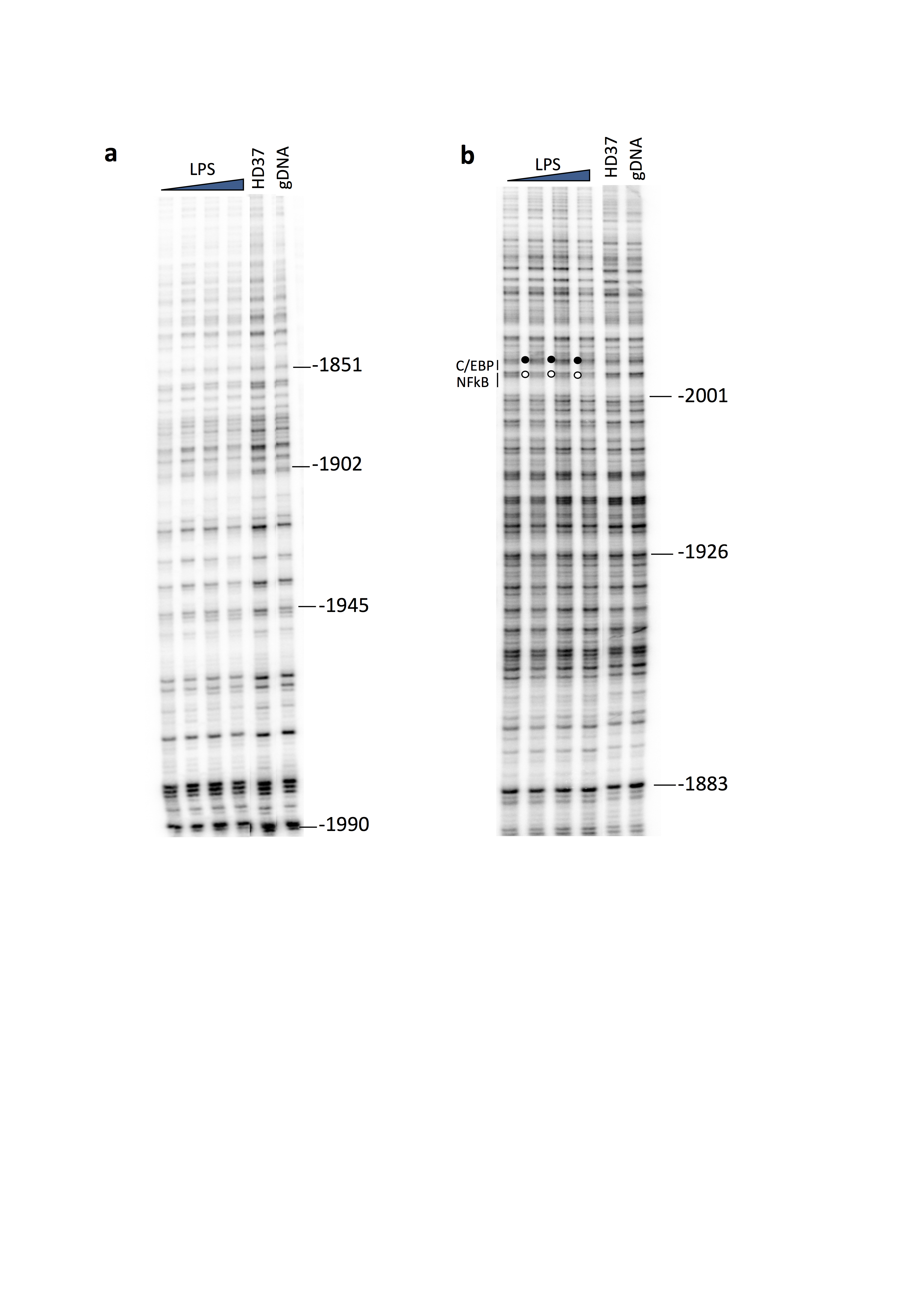

Supplement: Figure S1 — In vivo DMS footprinting of the distal part of the −1.9 kb promoter. HD11 cells were, in order from left to right, either unstimulated or LPS (1 µg/ml) stimulated for 30 min, 60 min or 240 min. Cells were then treated with DMS before the isolation of genomic DNA for hot piperidne cleavage and LM-PCR analysis. The HD37 erythroid cell line which do not express clys and the naked HD11 genomic DNA, G reaction, reference sequence are also shown (a) non-coding strand and (b) coding strand. The filled circles represent DMS hyper-methylation and the open circles base protection from DMS. The positions of the selected G bases are indicated relative to the clys transcription start site. The potential transcription factors are indicated adjacent to a single line encompassing their proposed binding site. These images are representative of two independent experiments. (TIFF) [file pone.0059389.s001.tiff]

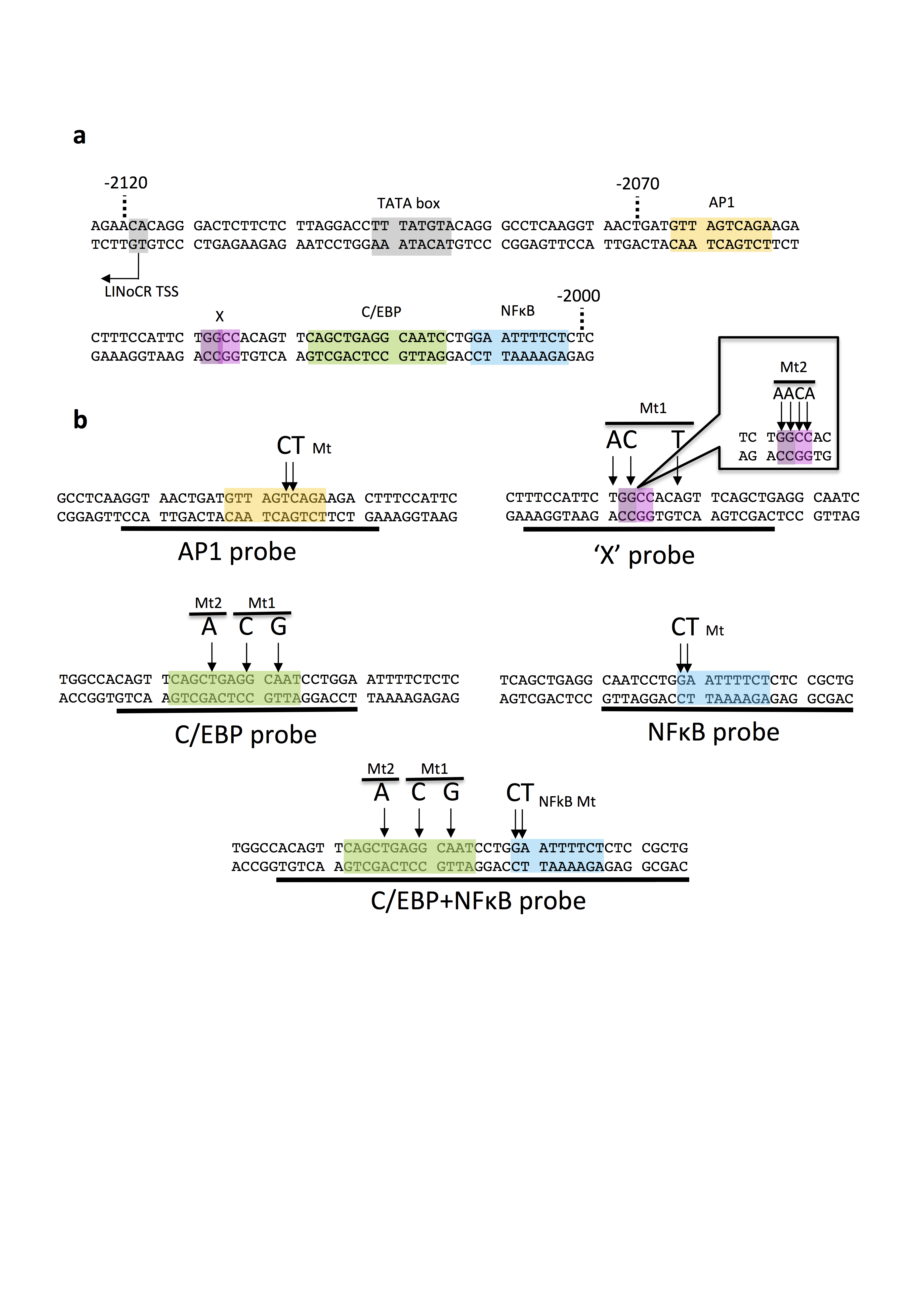

Supplement: Figure S2 — Sequence of the −1.9 Kb promoter/enhancer element and detailed EMSA’s probes. (a) The proposed transcription factor binding sites are double lined and colour coded; the AP1 site is gold, C/EBP site is green, NF-κB site is blue and DMS footprints for X (unidentified) are purple. LINoCR transcription start site (TSS) and proposed TATA box are shaded grey. Numbers located above the sequence are base pair positions relative to the clys transcription start site. (b) Probes designed for EMSA experiments. Base pair exchanges in designed mutants (Mt) are indicated at the top of the specific sequence. (TIFF) [file pone.0059389.s002.tiff]

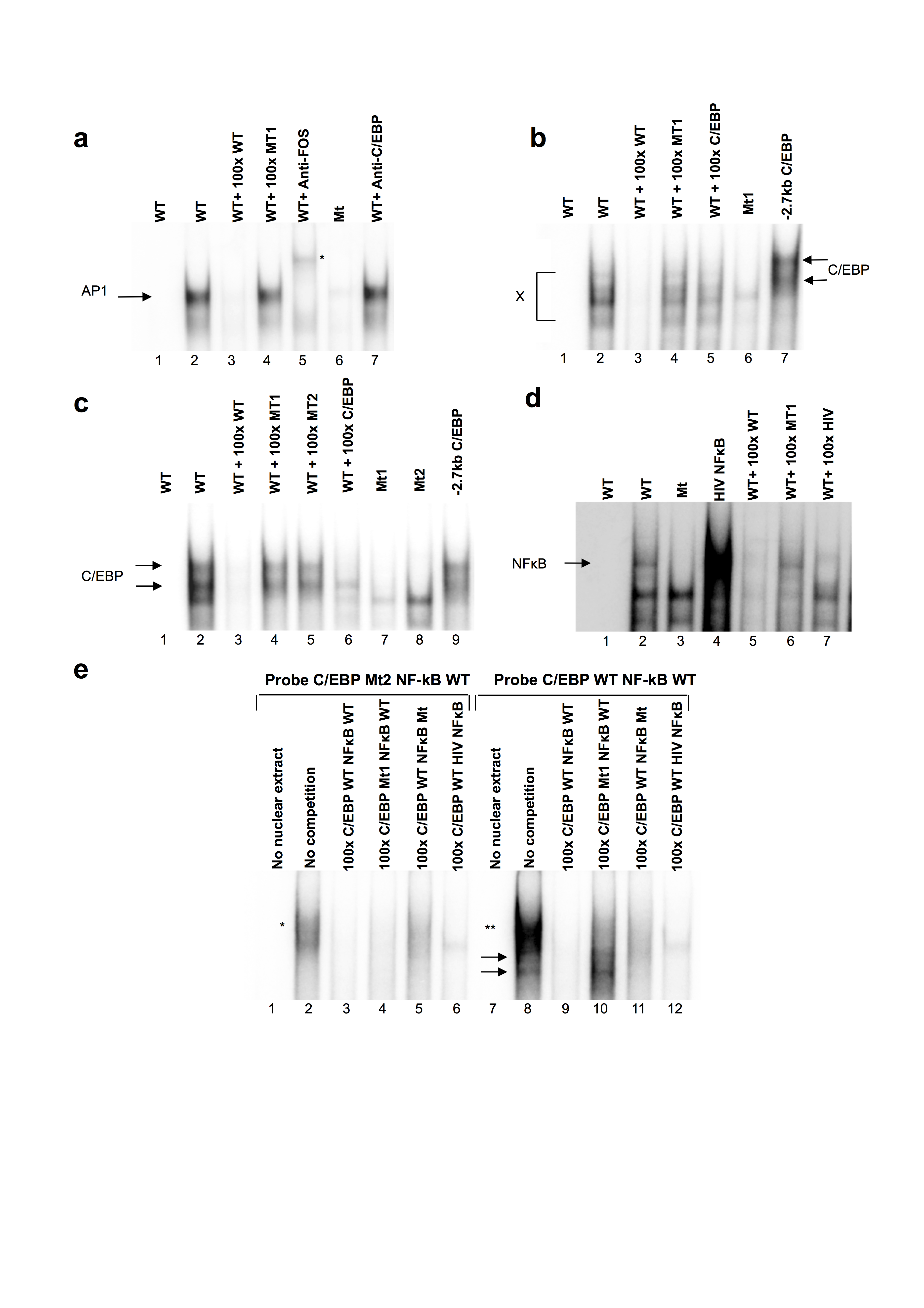

Supplement: Figure S3 — AP1, C/EBP and NF-κB transcription factors bind to the proposed site in the −1.9 kb CRE in vitro . Electromobility Shift Assay demonstrating specific binding of (a) AP1, (b) X (unidentified), (c) C/EBP, (d) NF-κB and (e) C/EBP and NF-κB to the −1.9 Kb element. 32P labelled oligonucleotide probes were incubated with crude nuclear extract from HD11 stimulated 1 hr with LPS (1 µg/ml) or with buffer alone (lanes 1). Specific DNA:Protein complexes are indicated with arrows. The supershift, when the reaction mixture was incubated with 1 µg of anti-cFos antibody (a, Lane 5), is indicated with an asterisk (*). Probes and cold competitors (100x) or antibodies (anti-) are indicated at the top of each lane. Sequences of the probes are detailed in figure S2. These figures are representative of two independent experiments. (TIFF) [file pone.0059389.s003.tiff]

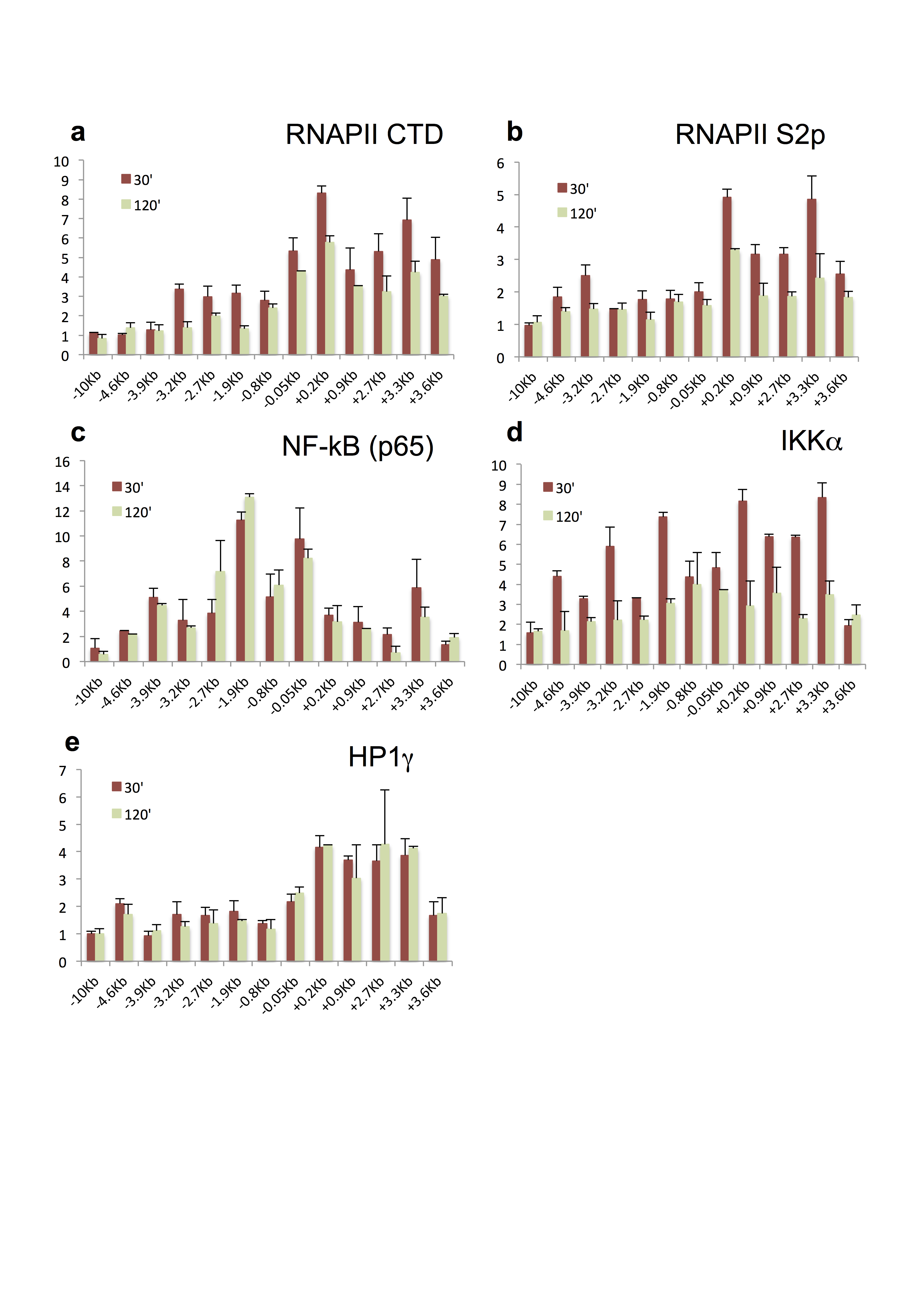

Supplement: Figure S4 — IKKα recruitment to the cLys locus is transient. (a–e) ChIP performed with primary macrophages treated with LPS for the indicated time points in minutes and the following antibodies (a) anti-RNAPII CTD, (b) anti-RNAPII S2p, (c) anti-p65 (NF-κB), (d) anti-IKKα and (e) anti-HP1γ. Horizontal axis indicates primers used for the Real time PCR (distance in Kb from the transcription start site of cLys). Data are normalized versus input and then versus a background control region designed within a CTCF binding site at the IL6 locus. Error bars represent SD from three independent qPCR replicates. These data are representative of at least three independent experiments. (TIFF) [file pone.0059389.s004.tiff]

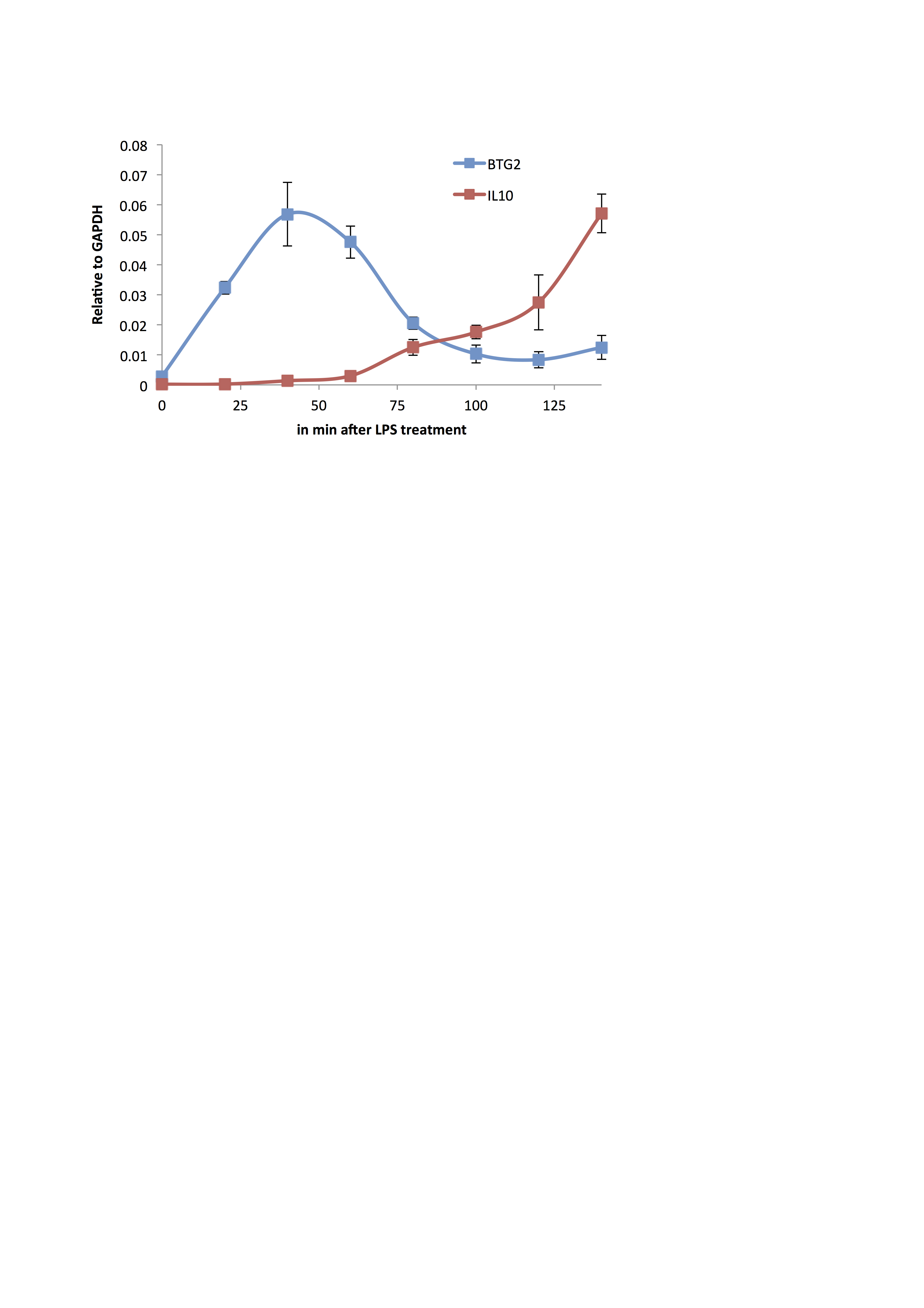

Supplement: Figure S5 — Changes in BTG2 and IL10 expression in response to LPS treatment. Time course of BTG2 (blue squares) and IL10 (red squares) mRNA levels in RAW264.7 cells in response to LPS treatment. Results are expressed relative to GAPDH expression. Error bars represent SD from three independent experiments. (TIFF) [file pone.0059389.s005.tiff]

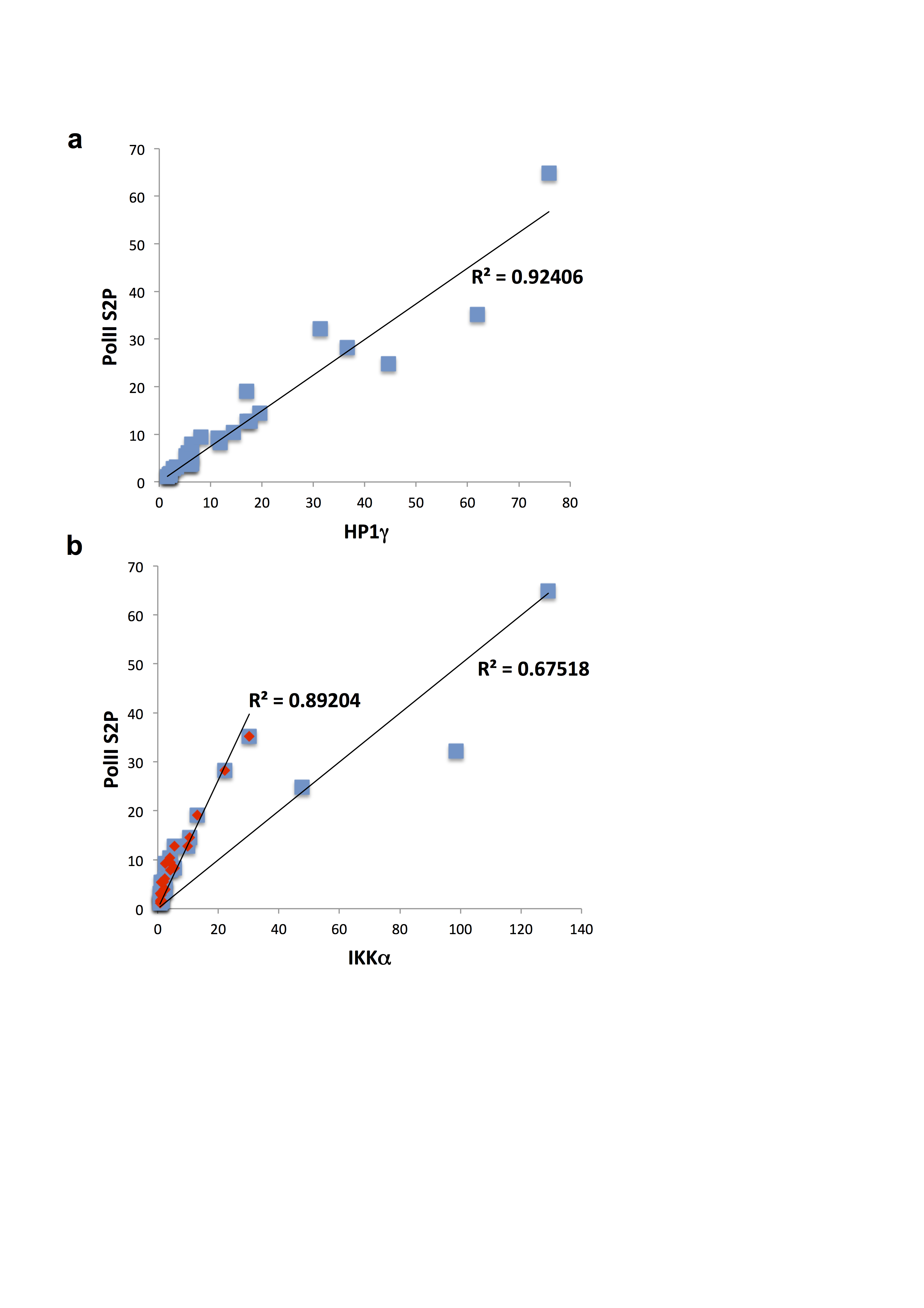

Supplement: Figure S6 — IKKα accumulates downstream of TNF TES independently of the elongating polymerase. (a and b) scatter plots showing the degree of correlation between the elongating polymerase (Y axis) and (a) HP1γ (x axis) or (b) IKKα (x axis). Blue rectangles display values from data presented in figure 6. Red lozenges are the same values without TNF TES 30′, 120′ and 240′. Trend lines and R-squared values are display on the figures. (TIFF) [file pone.0059389.s006.tiff]

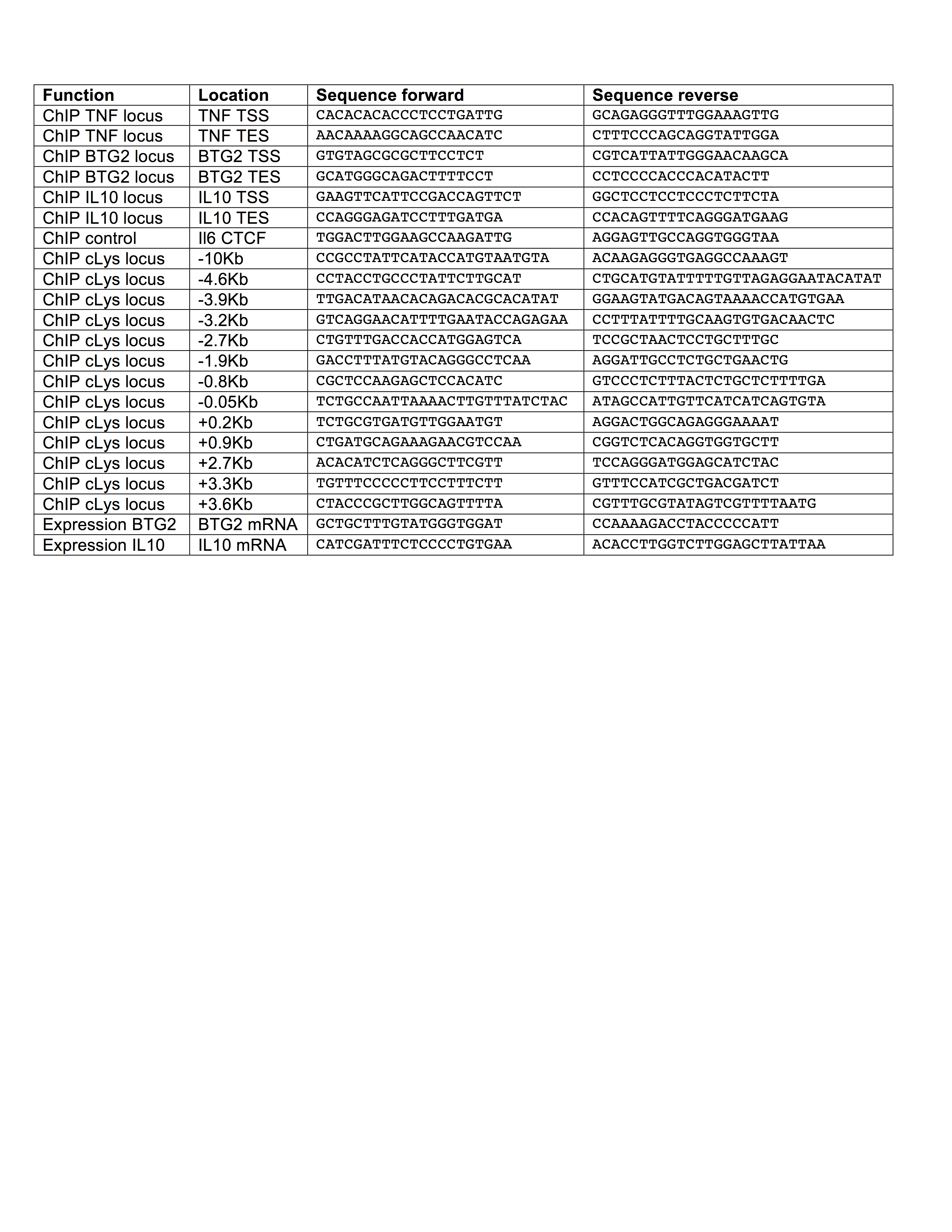

Supplement: Table S1 — List of primers used in this study. (TIFF) [file pone.0059389.s007.tiff]
